# Chloroform and the Dentists

**Published:** 1878-07

**Authors:** 


*Chloroform and the Dentists.*—"Mrs. Elizabeth Neely came to her death, March 20th, 1878, at No. 224 North Tenth Street, Philadelphia, by chloroform administered by Dr. H. G. Winslow, * * and Dr. Winslow is guilty of criminal ignorance in administering so powerful a remedy, and in not having made any examination of his patient."

So reads the verdict of the Coroner's jury. This case has gone the rounds of the press, and severe animadversions made upon the criminality of such conduct. Dr. Winslow has been stigmatized as little less than a murderer for using so dangerous an agent as chloroform at all, when the equally efficient and ennoxious nitrous oxide gas was available, or when ether might be used as well. Not a little abuse also has been made of the dental profession for daring to use chloroform, the newspaper critics seeming to think that the responsibility of administering this agent should be shouldered by a physician; though why, if the chloroform is to kill, the physician should bear the burden of the death, is hard to see; and why the educated dentist should not be able to select his cases for anæsthesia by chloroform, is equally difficult of comprehension. Certainly dentists *ought* to be posted on this subject if constant presentation of it in dental and other literature avails to enlighten them.

But it so happens in this case, that the administrator of the chloroform was not a dental but a medical man, and a medical graduate, who graduated in the Jefferson Medical College, in 1841, and had been since then engaged chiefly in the practice of medicine. He was looked upon rather as an expert in the administration of chloroform, and was frequently employed by surgeons to administer it for them. In the case in question he gave the anæsthetic without making an examination of the patient, which the coroner's physician, Dr. Chapman, testified should have been done, and

"He thought that before administering either chloroform or ether the physician or dentist should, on all occasions, make a careful examination, and ascertain whether or not any heart trouble existed. He thought deceased had been suffering from disease of the heart previous to having the chloroform administered by Dr. Winslow, as the clot adhering to the heart gave evidence of having been there for some time. From the flabby condition of the heart, the chloroform, in the opinion of witness, must have directly acted upon that organ; he thought that ether was less dangerous than chloroform; very few cases of death have resulted from the former, while hundreds of cases are recorded as having resulted fatally by the latter."

The testimony of Dr. Winslow was that the lady came to have some teeth extracted;

"He asked her whether or not she was well, and she answered yes; administered chloroform and extracted three teeth, she at the time being in a recumbent position; after extracting the three teeth she came to and complained of feeling the pain; administered more chloroform on a napkin, which he placed in close proximity to her nose, but not close enough to come in contact with the face, and she again sank into insensibility; in pulling an eye-tooth, after having administered the chloroform the second time, it broke off, and in attempting to remove the remaining portion, found that respiration had ceased with patient; applied the usual remedies, consisting of electricity, throwing water in face, and made every effort to secure an artificial respiration, but it was useless as she ceased breathing; witness summoned Drs. Jackson and Gross, but when they arrived she had expired; witness stated that he had used chloroform for a number of years, but never had anything serious to result from its use; he would not have administered it to Mrs. Neely if she had informed him that she was suffering from heart disease; the quantity of chloroform given to the deceased was about ½ an ounce; he has used both ether and nitrous oxide gas, and was of the opinion that there was as little danger in using chloroform as either of the former."

Other witnesses testified to the apparently healthy condition of the patient.

The flippancy with which coroners' juries decide the question of the relative harmlessness of anæsthetics, and the oracular tone in which the coroner in this case pronounces his "opinion" as to the guilt of the action of the administrators of such agents, is certainly remarkable. For a jury to decide that death was caused by the chloroform was advanced ground to take, considering that the patient had an "old clot in the heart," and considering that such cases are dropping about the streets of our cities every day, or falling dead in their houses; and it is surely more than a coroner or his advising physician can safely say to assure the public that under such circumstances ether was much safer, and nitrous oxide gas was *perfectly safe!*

What we wish specially to call attention to, is the fact that this case has been paraded over the country as the case of a dentist, and not a few harsh and unjust criticisms have been made on this assumption of a fact. There are ignorant dentists, as there are ignorant physicians, but it is hardly right to assume that in all cases the dentist is possessed of no ability to judge of danger in such cases, or to promptly decide upon the means of succor in case of an untoward result.

But the tide is turning away from anæsthetics, and it is well. Many dentists who gave gas have given up its use, and the tendency is strong toward the more or less complete abandonment of general anæsthesia, by this profession. Surely the day will yet come when a safe and efficient method of *local anœsthesia* will be discovered, and toward this end the studies of the dental profession should be turned. H.

